# Professional and ethical values in Nursing practice: An Indian Perspective

**DOI:** 10.17533/udea.iee.v39n2e12

**Published:** 2021-06-15

**Authors:** Vijayalakshmi Poreddi, Athira Narayanan, Athira Thankachan, Binila Joy, Changhorla Awungshi, S Sai Nikhil Reddy

**Affiliations:** 1 RN, RM, BSN.MSN, Ph.D. Clinical Instructor. Email: pvijayalakshmireddy@gmail.com pvijayalakshmireddy@gmail.com; 2 RN, RM, BSN, Former Nursing intern. Email: athiranarayanan15@gmail.com athiranarayanan15@gmail.com; 3 RN, RM, BSN, Former Nursing intern. Email: athirathankachan97@gmail.com athirathankachan97@gmail.com; 4 RN, RM, BSN, Former Nursing intern. Email: jbinila@gmail.com jbinila@gmail.com; 5 RN, RM, BSN, Former Nursing intern. Email: changhorlaawungshi@gmail.com changhorlaawungshi@gmail.com; 6 MBBS, Former medical intern, Bangalore Medical College and Research Institute, Bangalore, India. Email: saithereddy@gmail.com Bangalore Medical College and Research Institute Bangalore India saithereddy@gmail.com; 7 College of Nursing, National Institute of Mental Health and Neuro Sciences, (Institute of National Importance), Bangalore, India. National Institute Of Mental Health And Neurosciences National Institute of Mental Health and Neuro Sciences Bangalore India

**Keywords:** ethics, nursing, professionalism, social values, cross-sectional studies, India, ética en enfermería, profesionalismo, valores sociales, estudios transversales, India., ética em enfermagem, profissionalismo, valores sociais, estudos transversais, India.

## Abstract

**Objective.:**

This study was carried out to examine professional and ethical values related to the profession from nurses' perspectives.

**Methods.:**

This was a cross-sectional survey carried out among 124 randomly selected nurses working at a tertiary care hospital in South India. The data was collected using Nursing Professional Values scale (NPVS-3). This tool consisted of 28 items to assess nurses’ professional values in three domains namely; Caring (10 items), activism(10items), and professionalism (8items). The maximum range of scores is 28-140. The higher the score, the stronger the nurse’s professional value orientation.

**Results.:**

The mean total score of the Professional Value scale was high (121.07±15.32). The mean score of the participants was higher in the caring domain (44.02±5.75) than activism (42.19±6.33) and professionalism domains (34.86±4.27). Pearson correlational analysis revealed that nurses with less experience had greater mean professional values score than nurses with higher experience (*p*<0.01).

**Conclusion.:**

The present study showed that nurses have high professional and ethical values, although they perceive that the most important values are those related to direct patient care. Continuing education programs should be designed so that nurses understand that nonclinical professional values are also equally important in promoting the nursing profession.

## Resumen

**Objetivo.:**

Este estudio se llevó a cabo para evaluar los valores profesionales y éticos relacionados con la profesión desde la perspectiva de los enfermeros.

**Métodos.:**

Se trata de un estudio transversal realizado entre 124 enfermeras seleccionadas aleatoriamente quienes trabajan en un hospital de atención terciaria del sur de la India. Los datos se recogieron utilizando la escala de valores profesionales de enfermería (NPVS-3). Este instrumento consta de 28 ítems con respuesta tipo Likert de 1-5 que evalúa los valores profesionales de los enfermeros en tres dominios: cuidado (10 ítems), activismo (10 ítems) y profesionalismo (8 ítems). El rango máximo de las puntuaciones es de 28 a 140, cuanto mayor sea la puntuación, mayor será la orientación hacia los valores profesionales de enfermería.

**Resultados.:**

La puntuación media total de la escala de valores profesionales fue alta (121.07±15.32). La puntuación media de los participantes fue más alta en el dominio de cuidado (44.02±5.75) que en el del activismo (42.19±6.33) y en el de profesionalismo (34.86±4.27). El análisis correlacional de Pearson reveló que las enfermeras con menos experiencia tenían una mayor puntuación media en valores profesionales que las enfermeras con mayor experiencia (*p*<0.01).

**Conclusión.:**

El presente estudio mostró que las enfermeras tienen altos valores profesionales y éticos, aunque perciben que los valores más importantes son los relacionados con el cuidado directo al paciente. Deben diseñarse programas de formación continua para que las enfermeras comprendan que los valores profesionales no clínicos también son igualmente importantes para promover la profesión de enfermería

## Resumo

**Objetivo.:**

Este estudo foi realizado com o objetivo de avaliar os valores profissionais e éticos relacionados à profissão na perspectiva dos enfermeiros.

**Métodos.:**

Este é um estudo transversal de 124 enfermeiras selecionadas aleatoriamente que trabalham em um hospital terciário no sul da Índia. Os dados foram coletados por meio da Nursing Professional Values ​​Scale (NPVS-3). Esse instrumento é composto por 28 itens com resposta do tipo Likert de 1 a 5 que avaliam os valores profissionais do enfermeiro em três domínios: cuidado (10 itens), ativismo (10 itens) e profissionalismo (8 itens). A faixa máxima de pontuação é de 28 a 140, quanto maior a pontuação, maior a orientação para os valores do profissional de enfermagem.

**Resultados.:**

A pontuação média total da escala de valores profissionais foi elevada (121.07±15.32). A pontuação média dos participantes foi maior no domínio cuidar (44.02±5.75) do que no ativismo (42.19±6.33) e no domínio profissionalismo (34.86±4.27). A análise correlacional de Pearson revelou que enfermeiras menos experientes tiveram uma pontuação média mais alta em valores profissionais do que enfermeiras mais experientes (*p*<0.01).

**Conclusão.:**

O presente estudo mostrou que os enfermeiros possuem elevados valores profissionais e éticos, embora percebam que os valores mais importantes são aqueles relacionados ao cuidado direto ao paciente. Os programas de educação continuada devem ser planejados de forma que os enfermeiros entendam que os valores profissionais não clínicos também são igualmente importantes na promoção da profissão de enfermagem.

## Introduction

Nursing is one of the most trusted professions which is rooted in professional ethics and values.(1) Professional nursing values are defined as important professional nursing principles of human dignity, integrity, altruism, and justice that serve as a framework for standards, professional practice, and evaluation.(2) Values play a key role in any profession including the nursing profession. Professional values are articulated in the code of ethics. In India, every newly registered nurse is expected to adhere to the code of ethics and code of professional conduct for nurses developed by Indian Nursing council.

Ethics and professional values enlighten the nurses in providing quality of care to their clients. Furthermore, professional values not only influence individuals’ principles, behavior standards but also enable them in the creation of an ethical framework.(3,4) Weis and Schank argued that professional values are standards for behaviors that are recognized by professional groups and individuals, and are used to evaluate the integrity of the individual or organization.(5) However, according to the International Council of Nurses, the core nursing professional values include caring, activism, professionalism, trust, and justice.(6)

Published evidence report that professional values are vital components in nursing practice and nursing professionals need to be aware of these values as code of ethics to provide high-quality patient care.(6,7) Besides, professional values promote nurse’s ethical competency and their ability to manage ethical concerns. However, a majority of nurses were unaware of professional values and their relation to ethical issues.(8) Also, the code of ethics guides professional behaviors including the quality of professional care, patient safety, and the norms of the profession, and these values are crucial to professional practice and essential for developing and sustaining a professional identity.(9) Earlier research demonstrated that professional values among clinical nurses were high.(7,10) Further, most of the studies also have examined the nursing students’ views on the importance of professional values.(8,11) In India, very few studies have examined awareness of ethical principles among nurses^.^(12,13) However, research that focused on ethical and professional values among Indian nurses was limited. Therefore it is critical it is critical to investigate nurses’ knowledge of professional and ethical values and the integration of these values in their practice. Therefore, the present study was aimed to examine professional and ethical values related to the nursing profession from the perspective of nurses working in a tertiary care center.

## Methods

This was a cross-sectional survey carried out from November to December 2019 among nurses working at tertiary care hospital in South India. The sample was selected randomly using (random number table) nurses’ attendance register. The study criteria for nurses included having experience of more than one year and being directly involved in patient care. There were 156 nurses who were eligible to participate in this study and were invited for the same. Of the invited participants, nurses who were on leave (*n*=13), refused to participate (*n*=8) and few incomplete questionnaires (*n*=11) were excluded from the study. Therefore, the final sample size comprised of the present study was 124 nurses with 79% response rate.

The data was collected using a two-part questionnaire; the first part included demographic variables such as age, gender, religion, professional qualification, and professional experience and the second part included the Nursing Professional Values Scale-3 (NPVS-3) developed by Weis and Schank.(14) This tool consisted of 28 items to assess nurses’ professional values in three domains namely; Caring (10 items), activism(10 items), and professionalism (8 items). This was a Likert-scale rated from 1 (not important) to 5 (most important). The maximum range of scores is 28-140. The higher the score, the stronger the nurse’s professional value orientation. The Cronbach’s alphas are stated to be 0.942, with the subscale scores ranging from 0.70 to 0.85. Thus, the tool is considered valid and reliable.(14) In the present study, the reliability of the tool (r=0.86)was established through test-retest method among 20 nurses.

Data collection procedure. The English version of the NPVS-3 questionnaire was piloted among a small group (*n*=20) of nurses and found it was feasible. The researchers obtained permission from the Head of Department of Nursing and informed Nursing tutors and In-charge nurses about the study and requested a feasible time to administer the questionnaire among the selected nurses. Then the researchers met the nurses individually in their clinical postings and invited them to participate in the study. The questionnaires were distributed among the participants and collected back immediately. The primary researcher was available during the data collection to clarify the doubts of the participants if any. It took approximately 20-30 minutes to complete the questionnaires.

Ethical considerations. This study was approved by the Ethics committee at the College of Nursing and permission was obtained from the administrators of the hospital where the study was conducted. The nurses were informed of the study’s aims and procedures and obtained written informed consent from the volunteered participants. Data collection tools contained no identifying information to ensure the confidentiality of the participants. 

Statistical analysis. The data were analyzed using appropriate statistical software (SPSS 21 version) and results were presented in the form of tables. Descriptive statistics such as frequency, percentage, mean and standard deviation were performed. Inferential statistics such as Independent t-test and ANOVA were used to examine the correlation of professional values’ mean score with demographic variables of age, gender, religion, professional qualification, and professional experience. Pearson’s correlation coefficient was performed to investigate the correlation of the nursing professional values’ total score with the participants’ age. The level of significance was fixed at 0.05 level.

## Results

A total of 124 nurses have completed the NPVS-3 questionnaire. The mean age of the participants was 31.2years (7.06 SD). Most of the sample were females (70.2%), Hindus (54%) and graduates (75.8%) (Table 1). The majority of the participants had 6-15 years of experience in caring for the patients. According to Table 2 in NPVS-3 statements of nursing professional values, the statements “Respect the inherent dignity, values, and human rights of all individuals”, “Safeguard patient’s right to confidentiality and privacy” “Protect health and safety of the individuals” “Protect moral and legal rights of the patients” and “Accept responsibility and accountability for own practice” received the highest scores, while the statements “Engage in consultation and collaboration to provide optimal care”, “Take actions to influence legislatures and other policymakers to improve health care” “Participate in nursing research and/or implement research findings appropriate to practice” “Act as a patient advocate” “Confront practitioners with questionable or inappropriate practice” gained the lowest importance as the mean score was lesser.

**Table 1:** Relationship between nurses’ demographic characteristics and professional values

Variables	Frequency	Mean scores	***p*-value**
	***n* (%)**	(SD)	
Age			0.14
<25	32 (25.8)	125.34 (16.54)	
26-35	58 (46.8)	118.71 (14.40)	
>36	34 (27.4)	121.09 (15.25)	
Gender			0.37
Male	37 (29.8)	122.95 (14.95)	
Female	87 (70.2)	120.28 (15.50)	
Religion			0.89
Hindu	67 (54.0)	121.25 (15.25)	
Christian/others	57 (46.0)	120.86 (15.55)	
Professional qualification			0.98
GNM (Diploma in Nursing)	10 (8.1)	120.50 (13.98)	
BSc	94 (75.8)	121.23 (16.07)	
MSc	20 (16.1)	120.60 (12.77)	
Professional Experience years)			0.01
<5	53 (42.7)	125.15 (15.34)	
6-15	54 (43.5)	116.44 (14.97)	
16-25	17 (13.7)	123.06 (12.93)	

**Table 2:** Mean scores of Professional Values Scale (NPVS-3) statements

No	Statement	Mean (SD)
27	Engage in consultation and collaboration to provide optimal care	4.05 (0.98)
26	Take actions to influence legislatures and other policy makers to improve health care	4.05 (0.88)
17	Participate in nursing research and/or implement research findings appropriate to practice	4.15 (0.94)
16	Act as patient advocate	4.15 (0.98)
20	Confront practitioners with questionable or inappropriate practice	4.21 (0.83)
5	Participate in peer review	4.23 (0.75)
13	Assume responsibility for meeting health needs for diverse populations	4.24 (0.79)
11	Recognize the role of professional nursing associations in shaping health policy	4.26 (0.86)
25	Promote mutual peer support and collegial interactions to ensure quality care and professional satisfaction	4.26 (0.76)
28	Recognize professional boundaries	4.26 (0.84)
23	Actively promote health of the populations	4.27 (0.76)
21	Protect rights of participants in research	4.27 (0.83)
12	Establish collaborative partnerships to reduce health care disparities	4.28 (0.85)
24	Participate in professional efforts and collegial interactions to ensure quality care and professional satisfaction	4.29 (0.78)
22	Practice guided by principles of fidelity and respect	4.29 (0.89)
07	Promote and maintain standards where planned learning activities for students takes place	4.34 (0.77)
10	Advance the profession through active involvement in health-related activities	4.36 (0.83)
18	Provide care without bias or prejudice to patients and populations	4.36 (0.76)
06	Establish standards as a guide for practice	4.36 (0.74)
08	Initiate actions to improve environment of practice	4.41 (0.78)
01	Engage in on-going self-evaluation	4.45 (0.69)
04	Assume responsibility for personal well-being	4.46 (0.69)
09	Seek additional education to update knowledge and skills to maintain competency	4.46 (0.73)
15	Protect moral and legal rights of the patients	4.47 (0.76)
14	Accept responsibility and accountability for own practice	4.48 (0.73)
03	Protect health and safety of the individuals	4.56 (0.64)
19	Safeguard patient’s right to confidentiality and privacy	4.57 (0.68)
02	Respect the inherent dignity, values, and human rights of all individuals	4.65 (0.57)

The mean score of the participants was higher in the caring domain (44.02±5.75) than activism (42.19±6.33) and professionalism domains (34.86±4.27). These findings indicate that the participants perceived the professional values directly related to patient care as the most important. The mean total score of the professional values from the nurses’ perspectives was high (121.07±15.32) and suggests that 86% of the nurses in this study were aware of professional values in the nursing profession (Table 3).

**Table 3:** Subdimensions of Nursing Professional Values Scale (NPVS-3)

Domains of the scale	Range	Minimum Score	Maximum Score	Mean (SD)
Caring (10 items)	10 to 50	26	50	44.02 (5.75)
Activism (10 items)	10 to 50	26	50	42.19 (6.33)
Professionalism (8 items)	8 to 40	25	40	34.86 (4.27)
Total (28 items)	28 to 140	85	140	121.07 (15.32)

In this study, the mean professional value scores of age, gender, religion, and professional qualifications were not significantly different. However, a statistically significant difference observed between nurses’ professional experience and professional values scores. Nurses with less experience had greater mean professional values score than nurses with higher experience (*p*<0.01). Pearson’s correlational analysis revealed a positive relationship between caring, activism, and professionalism dimensions (*p*<0.001) (Table 4).

**Table 4:** Correlation between age and subdimensions of Nursing Professional Values Scale (NPVS-3)

Domains of the scale	Age	Caring	Activism
Caring	-0.074		
Caring	0.41		
Activism	-0.031	0.816	
Activism	0.73	0.001	
Professionalism	-0.014	0.813	0.817
Professionalism	0.87	0.001	0.001

## Discussion

This was the first study that explored the importance of professional and ethical values from the nurses’ perspective from Indian settings. The higher mean score of the professional values scale indicates that nurses in this study hold strong professional values in their practice. However, most of the participants perceived the professional values in the caring domain were most important than values stated in activism and professionalism domains. The fundamental responsibility of nurses is to provide safe, ethical, and quality of care. This can be achieved when the patients are treated with dignity, respect, and care and in this study, nurses rated high to the item “*Respect the inherent dignity, values, and human rights of all individuals”* with a mean score of 4.65(SD, 0.57).(15)

In line with previous research,(16) nurses in the present study rated caring domain items as most important professional values. These items include; “*Safeguard patient’s right to confidentiality and privacy” “Protect health and safety of the individuals” “Protect moral and legal rights of the patients” and “Accept responsibility and accountability for own practice”.* These findings can be attributed to the fact that nurses mainly consider the values that are directly related to their professional practice.(17) Furthermore, it can also be argued that nurses are involved in offering patient care even with significant changes in the health care system.(5) On the other hand, the items under activism and professionalism domains were low rated. The low rated items include; *“Engage in consultation and collaboration to provide optimal care”, “Take actions to influence legislatures and other policymakers to improve health care” “Participate in nursing research and/or implement research findings appropriate to practice” “Act as a patient advocate” “Confront practitioners with questionable or inappropriate practice”.* Similar findings were observed in other studies.(7,18) These findings could be due to nurses often are not actively involved in policymaking. Further, nurses may believe that these are non-clinical activities in the nursing practice. The mean value *“Participate in nursing research and/or implement research findings appropriate to practice”* was low as nurses are busy in providing patient care or maybe having negative perceptions such as 'difficult to understand the concepts of research.(19) Most importantly, nurses are expected to be ethical and patients rely on nurses to be advocates for them.(20) Therefore, nurse administrators need to consider these areas to improve professional values through CNE or in-service educational programs.

In this study, the mean total score of the professional values from the nurses’ perspective was high (121.07 ± 15.32) which suggests 86% of the nurses hold strong professional values. These results were consistent with findings of other studies conducted among nurses and the total mean score on professional values scale ranged 100.01 to 165.41.(16,21,22) These differences could be due to the attitudes and beliefs of nurses from various countries. Therefore, future studies should focus on the influence of culture on professional values among nurses.

Caring referred to as the core of nurses' professional values.(23) Similarly, most of the nurses in this study felt that the items in the caring domain as the main and most important professional values while the item ‘act as patient advocate ‘received the lowest score of 4.15±0.98. However, the mean score of the participants was higher in the caring domain than activism and professionalism domains. These findings indicate that the participants perceived the professional values directly related to patient care as the most important. Similar findings were observed in earlier studies.(16,18) Rabia S. Allari also stated that nursing issues outside the nurse-client relationship such as public policy, environment, professional nursing organizations, and research, and professional organizing were perceived as less important by nurses. Thus, it is an urgent concern to create awareness among nurses about the need to view the professional values under professionalism and activism domains as equally important to the caring domain to propel the profession to influence healthcare reform.

In this study, nurses with less experience had a greater mean professional value score than nurses with higher experience (*p*<0.01). These results were supported by findings in other studies carried out among nurses.(24) On the contrary, Poorchangizi *et al*. demonstrated that nurses with more experience have obtained higher scores of professional values. Similar to earlier research,(25) this study also indicates no statistically significant correlation between professional values scores and demographic variables (*p*>0.05). However, these findings were dissimilar to a study carried out among nurses from a developed country.^(26 )^Probably, these findings could be due to cultural differences.

Limitations. The present study has certain limitations such as the sample was selected from a single setting, a cross-sectional survey design, and the data was collected using self-reported questionnaires. Therefore, there may be a possibility of bias and exaggeration of scores. Due to these reasons generalization of the findings was limited. Further, the NPVS-3 scale measured the importance of professional values from the nurses’ perspective, but it did not assess the application of these values in nursing practice. Future studies should focus on mixed methods designs to understand nurses' views on ethical and professional values and ethical challenges they encounter while incorporating these values into professional practice. Despite these limitations, the findings would be helpful for nurse educators and administrators to develop continuous educational programs to improve nurses’ awareness and understanding of the importance of professional values and improve the quality of care.

## Conclusion

The present study shows that nurses hold high professional and ethical values which were prioritized from the nurses’ perspective as caring, activism and, professionalism, respectively. Most of the nurses perceived the professional values related to direct patient care are most important. On the other hand, professional values related to nurses engaging in ‘consultation and collaboration’, ‘policy-making’, and ‘evidence-based practice’ were perceived by nurses as less important. Therefore, nurse administrators need to emphasize the less-important professional values in designing continuous education programs to improve nurses' understanding that non-clinical professional values also are equally important to promote the nursing profession.
